# Progressive Unilateral Moyamoya-like Vasculopathy After Head Trauma with Chronic Subdural Hematoma: A Case Demonstrating the Utility of Anterior Circulation Basi-Parallel Anatomical Scanning

**DOI:** 10.3390/neurolint17120191

**Published:** 2025-11-26

**Authors:** Shinya Watanabe, Yasushi Shibata, Eiichi Ishikawa

**Affiliations:** 1 Department of Neurosurgery, Mito Kyodo General Hospital, Tsukuba University Hospital Mito Area Medical Education Center, 3-2-7 Miyamachi, Mito 310-0015, Ibaraki, Japan; yshibata@md.tsukuba.ac.jp; 2 Department of Neurosurgery, Institute of Medicine, University of Tsukuba, 2-1-1, Amakubo, Tsukuba 305-8576, Ibaraki, Japan; e-ishikawa@md.tsukuba.ac.jp

**Keywords:** Moyamoya syndrome, chronic subdural hematoma, head trauma, vasculopathy, BPAS, anterior circulation

## Abstract

**Background**: Moyamoya syndrome is a moyamoya-like cerebrovascular condition associated with an identifiable underlying condition. Although head trauma has historically been considered a possible contributing factor, it is currently excluded from the Japanese diagnostic criteria. We report a rare case of progressive unilateral moyamoya-like vasculopathy that developed on the ipsilateral chronic subdural hematoma (CSDH) following head trauma, with a decade-long imaging follow-up. Anterior circulation basi-parallel anatomical scanning (BPAS) provided unique insights into the progressive vessel narrowing beyond the vascular lumen, suggesting its potential utility in evaluating such rare vasculopathies. **Case Presentation**: A 40-year-old man developed a left-sided CSDH after head trauma and underwent burr hole drainage. Although his symptoms resolved, serial magnetic resonance angiography (MRA) over the subsequent 10 years revealed progressive stenosis of the left middle cerebral artery (MCA), ultimately culminating in an occlusion-like appearance. BPAS revealed moyamoya-like collateral vessels in the same hemisphere, a significant reduction in the outer diameter of the left MCA, supporting the presence of structural arterial wall changes that were not apparent on conventional MRA. Single-photon emission computed tomography revealed mildly reduced cerebral blood flow on the affected side, with a difference of less than 5% compared to non-affected side. He remained neurologically non-symptomatic, with no history of transient ischemic attacks or overt ischemic stroke. **Conclusions**: This case highlights a rare clinical course of progressive ipsilateral moyamoya-like vascular changes following head trauma and burr-hole drainage for CSDH, potentially indicating an association between head trauma, CSDH, and subsequent moyamoya-like collateral vessel development, warranting further investigation. The use of the anterior circulation BPAS contributed to the detection of structural arterial changes that were not apparent on conventional MRA, suggesting its potential utility in evaluating such vascular abnormalities.

## 1. Introduction

Moyamoya disease is a chronic cerebrovascular disorder characterized by progressive stenosis or occlusion of the terminal portion of the internal carotid artery (ICA) and the proximal portions of the anterior and middle cerebral arteries (MCA), accompanied by an abnormal vascular network known as “Moyamoya-like collateral vessels.” When similar vascular findings are observed in patients with known underlying conditions, the diagnosis is termed “Moyamoya syndrome” [1,2]. Previously, “head trauma” had been listed as a possible underlying cause of Moyamoya syndrome; however, the revised Japanese diagnostic criteria for Moyamoya disease [3] published in 2021 omitted this association, and a causal relationship is currently considered unlikely. Notably, case reports describing Moyamoya syndrome secondary to head trauma are extremely rare [4,5]. Since the 2021 removal, accumulating well-documented post-traumatic cases may provide important insights into the possible underrecognized subtypes of Moyamoya syndrome. A scientific statement in 2023 by the American Heart Association noted that head trauma, excluded by the Research Committee on Moyamoya Disease guidelines, is still debated as a potentially associated condition in Moyamoya syndrome [1]. Therefore, there may be no international consensus regarding this exclusion.

Here, we report a rare case of a middle-aged man who developed chronic subdural hematoma (CSDH) following head trauma, underwent burr-hole drainage, and subsequently developed progressive stenosis of the ipsilateral ICA distal segment to the MCA, potentially due to unilateral moyamoya syndrome. The use of Basi-Parallel Anatomical Scanning (BPAS) [6] enabled the visualization of fine cerebral vessels in distinguishing structural abnormalities or arterial wall changes in the anterior circulation from simple stenosis. Given the rarity of moyamoya syndrome observed through long-term post-traumatic follow-up and the detailed imaging follow-up, we believe that this case has significant clinical and academic value.

## 2. Case Description

**Figure 1:**
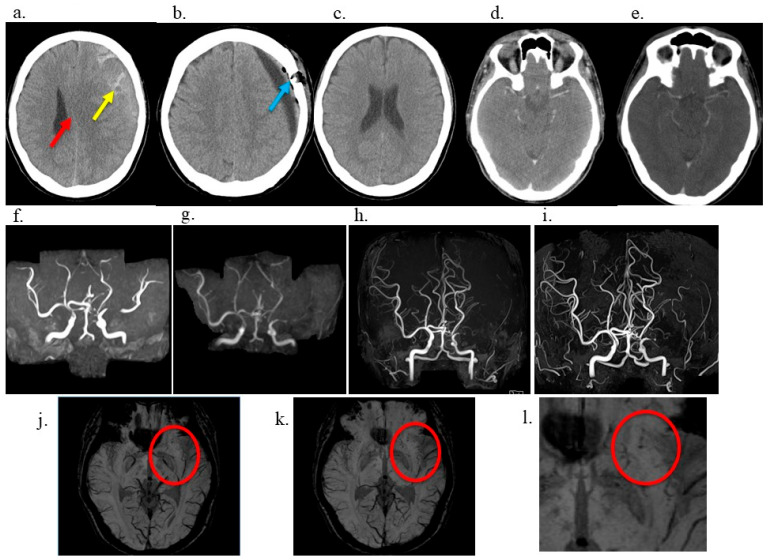
Head computed tomography (CT) or magnetic resonance imaging showing chronic subdural hematoma (CSDH) or progression of left middle cerebral artery (MCA) stenosis. (**a**) Preoperative head CT showing a left-sided CSDH (yellow arrow) with midline shift (red arrow). (**b**) Postoperative day 1 CT demonstrating resolution of the hematoma with adequate drainage (drain tube and burr hole: bule arrow) and improved midline shift. (**c**) CT obtained 1 month following surgery showing complete resolution of the hematoma and normalized brain contour. (**d**) Reference contrast-enhanced CT at 37 years old before CSDH showing symmetrical bilateral middle cerebral arteries (MCAs) without stenosis. (**e**) Contrast-enhanced CT at 41 years old, 1 year after CSDH, demonstrating no stenosis of the left MCA. (**f**) MRA at 43 years old, 3 years after CSDH, demonstrating mild stenosis of the left MCA. (**g**) MRA at 46 years old, 6 years after CSDH, showing significant progression of left MCA stenosis. (**h**) MRA at 47 years old, 7 years after CSDH, revealing non-visualization of the left MCA. (**i**) MRA at 50 years old, 10 years after CSDH, confirming continued non-visualization of the left MCA. (**j**,**k**) SWI demonstrating Moyamoya-like collateral vessels (red elliptical) in the left cerebral hemisphere suggestive of Moyamoya-like vascular networks. (**l**) Magnified SWI view corresponding to panel (**k**), demonstrating abnormal fine collateral vessels (red elliptical) in the left basal ganglia. These findings are characteristic of Moyamoya-like vasculopathy and were not observed on the contralateral side.

A 40-year-old male government employee sustained a head trauma while playing soccer. Approximately 2 months later, he experienced a gradually worsening headache and presented to our hospital. Head computed tomography (CT) revealed left-sided CSDH (Figure 1a), prompting admission. The patient had a medical history of right inguinal hernia repair, right-hand fracture, and papillary thyroid carcinoma at the ages of 10, 16, and 37 years, respectively, for which he underwent surgical resection only, with no radiation therapy, resulting in cure. Importantly, the patient had no prior history of radiotherapy, which is a known risk factor for moyamoya-like vasculopathy. Similarly, he had no history of hyperthyroidism. At 40 years of age, he was diagnosed with mild hypertension and treated with amlodipine monotherapy. Moreover, he had a smoking history of 10 cigarettes per day from the age of 17 to 38 years and occasionally consumed approximately one bottle of beer. There was no family history of Moyamoya disease or other cerebrovascular disorders. The patient underwent left burr hole irrigation and drainage on admission. His headache resolved promptly, and his CSDH was sufficiently drained (Figure 1b). He was discharged home 1 week later with a modified Rankin Scale (mRS) score [7] of 0, and no CSDH was observed on postoperative month 1 (Figure 1c). After surgery for CSDH, the patient was relocated to work and continued to follow up at another hospital. A review of contrast-enhanced CT performed at the age of 37 years for the evaluation of a thyroid nodule 3 years before the head trauma demonstrated a symmetrical depiction of the MCA, with no evidence of vascular abnormalities (Figure 1d); additionally, imaging performed at 41 years of age (1 year after the CSDH) exhibited no evidence of MCA stenosis (Figure 1e). However, magnetic resonance angiography (MRA) revealed mild stenosis of the left MCA at the age of 43 years (3 years since CSDH) (Figure 1f), which gradually progressed over time. The stenosis had become severe at the age of 46 (6 years from CSDH) (Figure 1g). Moreover, at the age of 47 (7 years from CSDH) (Figure 1h,i), the left MCA was no longer visible on MRA, a finding that persisted at the age of 50 (10 years from CSDH). Susceptibility-weighted imaging (SWI) demonstrated multiple fine, punctate, and linear low-signal structures in the left basal ganglia (Figure 1j–l), consistent with characteristic of Moyamoya-like collateral vessels.

**Figure 2:**
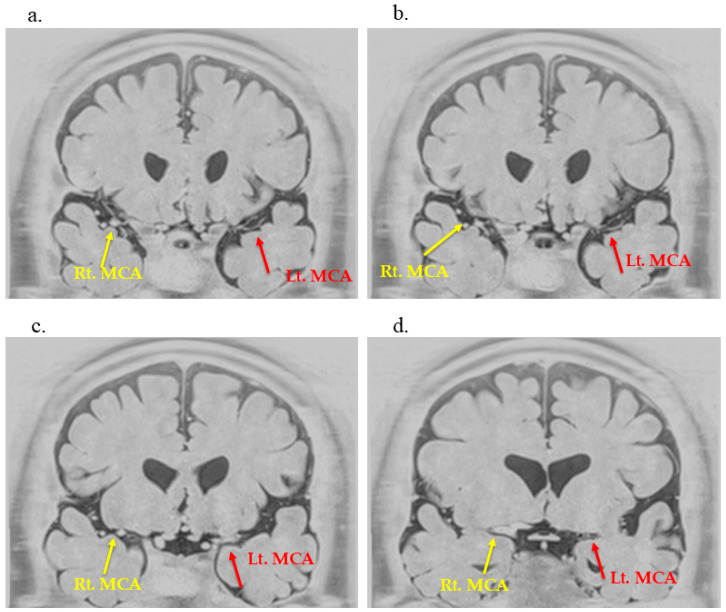
**Anterior circulation basi-parallel anatomical scanning (BPAS) reveals unilateral arterial outer diameter narrowing.** (**a**–**d**) BPAS imaging demonstrates marked reduction in the left MCA outer diameter (red arrows) compared with the right side (yellow arrows). By the final time point, the affected segment becomes poorly visualized on time-of-flight MRA (**d**), suggesting arterial wall involvement beyond simple luminal stenosis.

At the age of 53 years (13 years from CSDH), the patient remained neurologically stable, with an mRS score of 0 and no history of transient ischemic attacks or overt ischemic stroke. Resting single-photon emission computed tomography demonstrated a mild reduction (<5%) in cerebral blood flow on the affected side, which is evaluated by comparing the affected hemisphere with the contralateral side. Longitudinal SPECT studies performed repeatedly over the past five years demonstrated that this degree of mild hypoperfusion remained stable without interval deterioration. Additionally, to assess the arterial wall structure of the anterior circulation, BPAS of the anterior circulation was performed using the 3-Tesla MRI scanner (MAGNETOM Skyra, Siemens Healthineers, Erlangen, Germany) with a 20-channel head coil. The imaging parameters were as follows: slice thickness = 0.7 mm, coil type; head-neck 20 channels, field strength; 3.0 T, and post-processing software; none (Figure 2). Notably, the left MCA appeared remarkably thinner than the contralateral side, indicating a significant reduction in its outer diameter (Supplementary Figure S1(1-a): BPAS image, Figure S1(1-b): 2D TOF-MRA). These structural changes were not apparent on conventional time-of-flight (TOF)-MRA, underscoring the added value of BPAS for detecting subtle arterial wall abnormalities that may be difficult to distinguish from simple luminal stenosis on TOF-MRA alone.

The patient expressed surprise at the progressive nature of the vascular findings but was relieved to have remained neurologically intact and able to maintain his occupational and daily activities without restriction. Written informed consent was obtained from the patient for the publication of this case report and the accompanying images.

## 3. Discussion

Moyamoya syndrome is diagnosed in individuals who exhibit moyamoya-like cerebrovascular changes in association with an identifiable underlying condition [3,8]. The present case is extremely rare because it involved unilateral progressive stenosis of the MCA and the development of abnormal vascular networks observed long-term following head trauma and surgical intervention for CSDH. Notably, BPAS was crucial in detecting outer diameter narrowing of the anterior circulation vessels, particularly the left MCA, which were not clearly visualized on conventional TOF-MRA, highlighting the complementary value of anterior circulation BPAS [9,10] in evaluating subtle arterial wall changes and structural abnormalities in moyamoya-like conditions.

### 3.1. Previous Context Reported and Uniqueness of This Case

Previous reports have described CSDH occurring in patients with underlying moyamoya disease [11,12,13]. However, in those cases the moyamoya angiopathy pre-existed and the CSDH was secondary. In contrast, our case developed progressive unilateral moyamoya-like vasculopathy after head trauma and CSDH, suggesting a different pathophysiological mechanism.

Only two similar conditions were reported, including a pediatric case reported by Fernandez-Alvarez et al. in 1979, in which Moyamoya disease developed 3 years after head trauma [4], and another case reported by Zaletel et al. in 2011, in which Moyamoya changes were observed 24 years after traumatic injury [5]. Nonetheless, in both cases, the extended interval between the initial trauma and vascular pathology onset limits the ability to draw a strong causal inference. Conversely, our case demonstrated progressive vascular changes over a relatively short period following ipsilateral burr hole drainage for CSDH. Although digital subtraction angiography was not performed, Moyamoya-like collateral vessels were observed on SWI, which have been reported to reflect moyamoya-like vascular networks in previous studies [14]. Additionally, anterior circulation BPAS [9,10] demonstrated a remarkably thinner left MCA compared with the contralateral side, indicating a significant reduction in its outer diameter. Therefore, post-traumatic inflammation or alterations in cerebral hemodynamics may contribute to pathological vascular remodeling.

### 3.2. Potential Etiologies and Risk Factors

#### 3.2.1. Overview of Possible Mechanisms

Several pathophysiological mechanisms may be considered. Cigarette smoking has been reported as a potential contributor to intracranial large artery stenosis, particularly involving the ICA. Nevertheless, the reduction in outer diameter and the development of moyamoya-like collaterals and progressive M1 stenosis limited to the ipsilateral side following trauma suggests that additional factors beyond smoking may have contributed to the pathophysiology of this case.

#### 3.2.2. Endothelial Injury Hypothesis

One possibility may be the endothelial injury hypothesis, in which trauma-induced damage to the vascular endothelium triggers chronic inflammation and progressive stenosis. Although traumatic intracranial artery dissection can cause focal stenosis or occlusion, it typically presents with abrupt vascular narrowing, pseudoaneurysm formation, or intimal flap, most commonly affecting the proximal M1 segment. Although the progressive stenosis appeared three years from burr-hole drainage, the anatomical distance between the burr-hole site and the stenotic MCA segment suggests that a direct mechanical effect of the procedure is unlikely. However, given the single-case nature of this report, a causal relationship cannot be excluded, and further accumulation of similar cases is needed to clarify whether postoperative or post-traumatic factors contribute to unilateral moyamoya-like vasculopathy. This case demonstrated gradual, long-segment progressive narrowing from the distal ICA to the MCA over a decade, without imaging findings suggestive of dissection such as vessel wall irregularity, aneurysmal dilatation, or mural hematoma. Furthermore, BPAS imaging demonstrated that the reduction involved both the luminal narrowing and an actual decrease in the outer diameter of the vessels. These findings collectively may support the diagnosis of moyamoya-like vasculopathy rather than post-traumatic arterial dissection.

#### 3.2.3. Hemodynamic Redistribution Hypothesis

The hemodynamic redistribution hypothesis may indicate that trauma-induced changes in cerebral perfusion promote vascular remodeling and collateral formation. Previous reviews indicated that alterations in cerebral perfusion and chronic low-flow states can promote vascular remodeling, collateral vessel formation, and structural changes in the arterial wall in moyamoya angiopathy [15]. Rapid progression of steno-occlusive changes due to impaired collateral maturation and hemodynamic imbalance has also been described in rapidly progressive moyamoya presentations [16].

Such hemodynamic vulnerability and progressive steno-occlusive change have also been reported in adult-onset moyamoya syndrome, supporting the concept that flow redistribution and impaired reserve may accelerate disease progression [17]. Despite the progressive stenosis of the left MCA in this case, the patient remained neurologically asymptomatic. This clinical stability can be explained by the hemodynamic findings: the hemispheric cerebral blood flow difference on SPECT was less than 5%, a range generally considered physiologically acceptable and not typically associated with neurological deficits. Moreover, repeated SPECT examinations over the past 4–5 years demonstrated that this mild hypoperfusion remained stable without interval deterioration.

#### 3.2.4. Chronic Inflammation Hypothesis

The third possibility may be the chronic inflammation hypothesis, whereby a sustained inflammatory environment resulting from CSDH stimulates cytokine-mediated vascular changes. Endothelial dysfunction and chronic inflammatory signaling have been described as key pathogenic components of moyamoya angiopathy [15]. These observations suggest preserved hemodynamic compensation, likely supported by collateral circulation via the anterior or posterior communicating arteries. In clinical practice, when conventional TOF-MRA begins to show luminal attenuation or non-visualization in post-traumatic patients, performing anterior circulation BPAS [9,10] prior to invasive angiography may help differentiate true luminal narrowing from reduced vessel outer diameter and allow for non-invasive longitudinal monitoring.

The patient had a history of thyroid papillary carcinoma. Elevated thyroid autoantibodies and thyroid function are independently associated with Moyamoya disease [18]. Recent studies have suggested that RNF213, a known susceptibility gene for Moyamoya disease in East Asian populations, may also have immunological functions, including antimicrobial defense, via the ubiquitination of bacterial lipopolysaccharide [1,19]. These findings suggest a potential link between genetic predisposition and the immune-related pathophysiology of Moyamoya syndrome. Furthermore, autoimmune thyroid diseases, particularly the presence of thyroid autoantibodies such as anti-thyroid peroxidase and anti-thyroglobulin, have been reported to be significantly associated with moyamoya-like vasculopathy [20,21]. Although our patient had no clinical hyperthyroidism, the history of thyroid carcinoma warrants consideration of potential immune involvement. Despite no clinical evidence of hyperthyroidism, the possibility remains that autoimmune thyroid factors or subclinical thyroid dysfunction may have contributed to the disease pathogenesis. Evaluation of thyroid autoantibodies and thyroid function may help clarify this association.

### 3.3. Utility of BPAS in Detecting Moyamoya-like Vascular Changes

Although digital subtraction angiography (DSA) remains the gold standard for evaluating intracranial vasculopathy [22], it provides limited information regarding the external vessel contour. Recent reviews [23] highlighted that moyamoya angiopathy is characterized not only by luminal stenosis but also by progressive reduction in the outer diameter of affected arteries, underscoring the value of imaging modalities capable of depicting vessel wall morphology. BPAS offers a non-invasive, flow-independent technique that uniquely visualizes the outer boundaries of intracranial arteries, enabling distinction between true luminal narrowing and vessel wall shrinkage—features that are often difficult to appreciate on conventional TOF-MRA or DSA [2]. Nonetheless, anterior circulation BPAS [6,9,10] provided a clear delineation of the outer contours of the left MCA, revealing a notable reduction in outer diameter and confirming the presence of subtle yet significant vascular narrowing (Supplementary Figure S1(1-a): BPAS image, Figure S1(1-b): 2D TOF-MRA). BPAS imaging may be particularly advantageous for detecting changes in the vessel wall and identifying hypoplastic or structurally compromised arteries that may be difficult to evaluate with conventional luminal imaging techniques, such as TOF-MRA. In this case, BPAS allowed for the detection of fine collateral vessels and better characterization of the affected segment, potentially indicating a Moyamoya-like vasculopathy diagnosis rather than simple atherosclerotic stenosis. Because BPAS visualizes the outer contour of the vessel wall using a T2-weighted 3D fast spin-echo sequence independent of flow signal, it is less influenced by partial volume effects compared with TOF-MRA, thereby allowing a more reliable assessment of true vessel wall narrowing, the ability of BPAS to complement MRA by highlighting extracranial vessel morphology may offer important diagnostic value, especially in atypical cases such as unilateral Moyamoya syndrome or secondary vascular changes following trauma or surgery. Therefore, this BPAS imaging approach may aid in early detection, differentiation, and longitudinal monitoring of Moyamoya-like cerebrovascular changes.

### 3.4. Limitations

Although genetic testing for RNF213 variants was not performed in this case due to facility limitations, genetic testing for RNF213 variants [24], which are strongly associated with moyamoya disease in East Asian populations, may provide insights into any underlying genetic predisposition. Here, no family history or bilateral vascular changes were observed. Further accumulation of similar cases is essential to better understand the pathophysiological mechanisms and clinical significance of trauma-associated moyamoya-like vasculopathy.

This case report was prepared according to the CAse REport (CARE) guidelines [25] to ensure transparency and completeness of clinical reporting.

## 4. Conclusions

This case highlights a rare clinical course of progressive unilateral Moyamoya-like vascular changes following head trauma and burr-hole drainage for CSDH. The temporal sequence and radiological findings warrant further attention. Notably, the use of anterior circulation BPAS may facilitate the detection of structural arterial changes not apparent on conventional MRA, indicating its potential utility in evaluating such vascular abnormalities.

## Data Availability

The original contributions presented in the study are included in the article and Supplementary Material, further inquiries can be directed to the corresponding author.

## Supplementary Materials

The following supporting information can be downloaded at https://www.mdpi.com/article/10.3390/neurolint17120191/s1: Supplementary Figure S1: BPAS (1-a) and 2D TOF-MRA (1-b) illustrating the same vascular segment (left M1) on both modalities to highlight differences in outer vessel contour visibility.

## Abbreviations

The following abbreviations are used in this manuscript:

BPAS	Basi-parallel Anatomic Scanning magnetic resonance imaging
CARE	Case Report
CSDH	Chronic Subdural Hematoma
CT	Computed Tomography
ICA	Internal Carotid Artery
MCA	Middle Cerebral Artery
MRA	Magnetic Resonance Angiography
TOF	Time-of-Flight
mRS	Modified Rankin Scale
SWI	Susceptibility-Weighted Imaging
