# Supplementary figures and images for: Progressive Unilateral Moyamoya-like Vasculopathy After Head Trauma with Chronic Subdural Hematoma: A Case Demonstrating the Utility of Anterior Circulation Basi-Parallel Anatomical Scanning
Source: Neurol Int. 2025 Nov 26;17(12):191. doi: 10.3390/neurolint17120191 (PMC12736143; doi:10.3390/neurolint17120191)

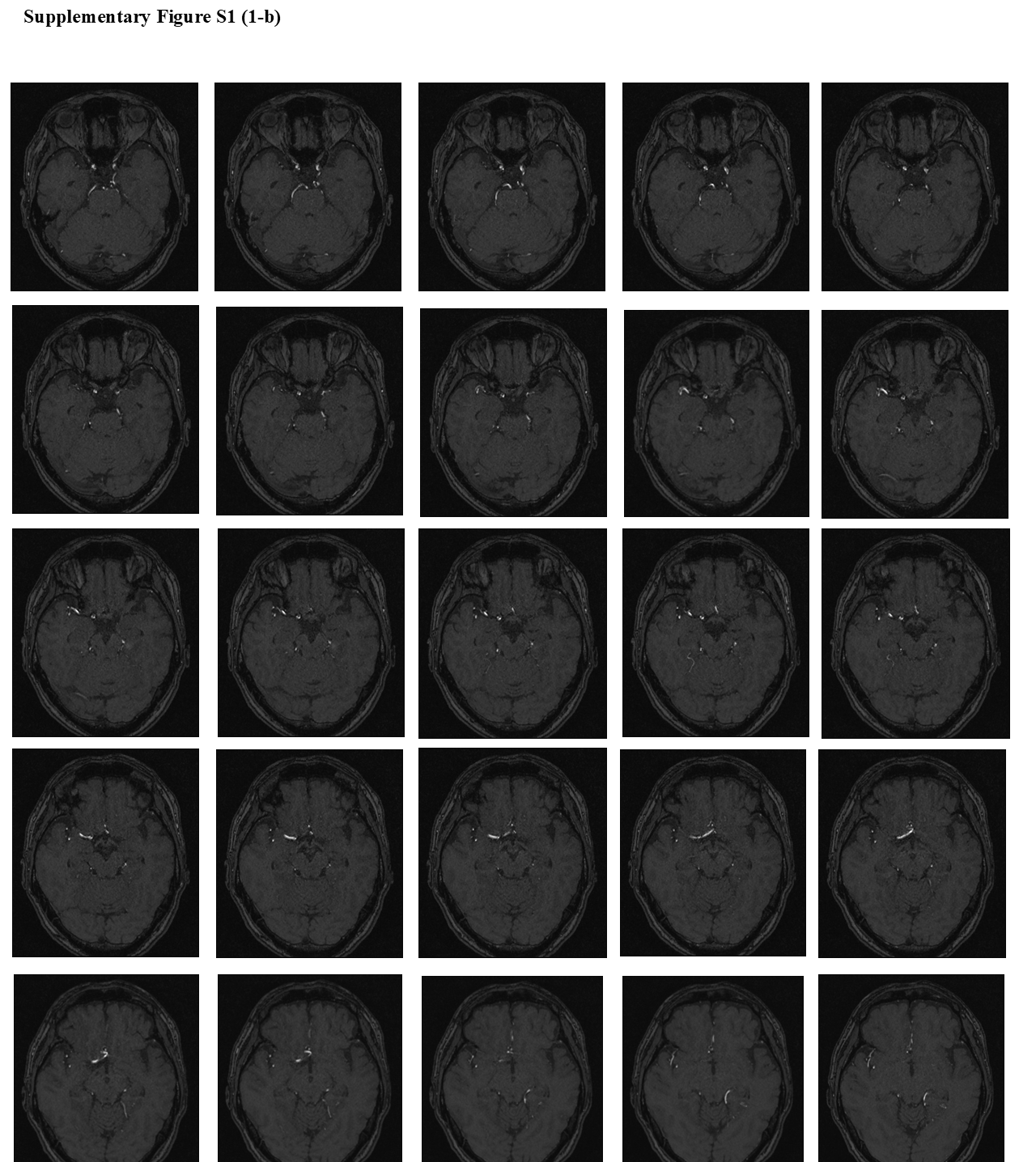

Supplement: Supplementary file 1 [file neurolint-17-00191-s001.zip › Supplementary Figure S1 (1-b).TIF]

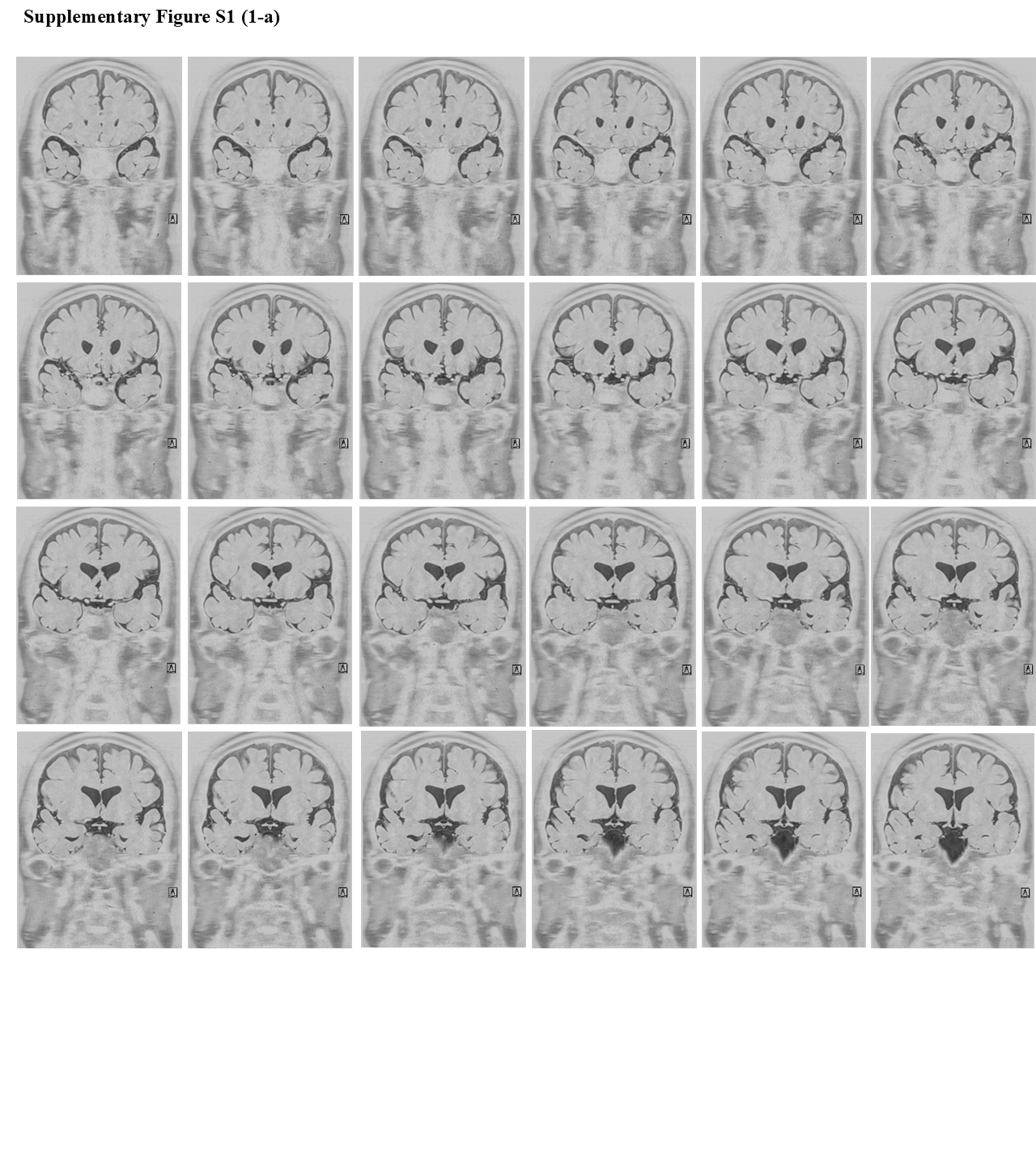

Supplement: Supplementary file 1 [file neurolint-17-00191-s001.zip › Supplementary Figure S1 (1-a).TIF]
